# Feasibility and acceptability of incorporating social network visualizations into a culturally centered motivational network intervention to prevent substance use among urban Native American emerging adults: a qualitative study 

**DOI:** 10.1186/s13722-022-00334-1

**Published:** 2022-09-30

**Authors:** David P. Kennedy, Elizabeth J. D’Amico, Ryan A. Brown, Alina I. Palimaru, Daniel L. Dickerson, Carrie L. Johnson, Anthony Lopez

**Affiliations:** 1 RAND Corporation, 1776 Main St., 2138, Santa Monica, CA 90401 USA; 2 UCLA Integrated Substance Abuse Programs, Semel Institute for Neuroscience and Human Behavior David Geffen School of Medicine, 11075 Santa Monica Blvd., Ste. 200, Los Angeles, CA 90025 USA; 3 Sacred Path Indigenous Wellness Center, Los Angeles, CA 90017 USA

**Keywords:** Native Americans, Social networks, Motivational Interviewing, Substance use, Personal network visualizations, Emerging Adults, Qualitative, Alcohol and other drug use, EgoWeb 2.0

## Abstract

**Background:**

Coupling social network visualizations with Motivational Interviewing in substance use interventions has been shown to be acceptable and feasible in several pilot tests, and has been associated with changes in participants’ substance use and social networks. The objective of this study was to assess acceptability and feasibility of an adaptation of this behavior change approach into a culturally centered behavior change intervention for American Indian/Alaska Native (AI/AN) emerging adults living in urban areas. AI/AN populations experience high rates of health disparities and substance use. Although 70% of AI/AN people live outside of tribal lands, there are few culturally tailored health interventions for these AI/AN populations. Social networks can both increase and discourage substance use. Leveraging healthy social networks and increasing protective factors among urban AI/AN emerging adults may help increase resilience.

**Methods:**

We conducted thirteen focus groups with 91 male and female participants (32 urban AI/AN emerging adults ages 18–25, 26 parents, and 33 providers) and one pilot test of the three workshop sessions with 15 AI/AN emerging adults. Focus group participants provided feedback on a proposed workshop-based intervention curriculum that combined group Motivational Interviewing (MI) and social network visualizations. Pilot workshop participants viewed their own social networks during group MI sessions focused on substance use and traditional practices and discussed their reactions to viewing and discussing their networks during these sessions. We used a combination of open coding of focus group and workshop session transcripts to identify themes across the group sessions and content analysis of comments entered into an online social network interview platform to assess the extent that participants had an intuitive understanding of the information conveyed through network diagrams.

**Results:**

Focus group and pilot test participants reacted positively to the intervention content and approach and provided constructive feedback on components that should be changed. Themes that emerged included feasibility, acceptability, relevance, understandability, and usefulness of viewing personal network visualizations and discussing social networks during group MI workshops. Workshop participants demonstrated an intuitive understanding of network concepts (network composition and structure) when viewing their diagrams for the first time.

**Conclusions:**

Social network visualizations are a promising tool for increasing awareness of social challenges and sources of resilience for urban AI/AN emerging adults. Coupled with Motivational Interviewing in a group context, social network visualizations may enhance discussions of network influences on substance use and engagement in traditional practices.

*Trial Registration*: ClinicalTrials.gov Identifier: NCT04617938. Registered October 26, 2020

## Background

Numerous studies have described health disparities among American Indian and Alaska Native (AI/AN) people due to colonization, forced relocation, and federal policies focused on assimilation and destruction of AI/AN culture. These disparities include high rates of homelessness, unemployment, poverty, poor mental health [1–7], and significant alcohol and other drug use (AOD) problems [8, 9]. One outcome of these federal policies is that many AI/AN families had to relocate to urban areas, which decreased connection to culture and traditions [10, 11] as many urban areas are geographically and socially fragmented [12]. To date, more than 70% of AI/AN people live outside of reservations and tribal lands [13, 14]. Despite the negative effects of these policies on health and well-being of the AI/AN population, many studies have highlighted the resilience of AI/AN people [e.g., 15] and the fostering of supportive social networks to help prevent the onset of substance use among this population.

One of the most pressing health issues for AI/AN people is alcohol and drug use. Recent data show that opioid use has reached epidemic proportions among AI/AN people [16–18]. Of particular concern is the increase in alcohol, cannabis and opioid use [19]—as well as substance use disorders [20]—among all emerging adults (ages 18–25), including AI/AN emerging adults. This is alarming due to the heightened vulnerability and critical social, neurological, and psychological development during this developmental period [21, 22]. Data from the National Survey on Drug Use and Health (NSDUH) in 2019 indicate that 45% of Native American emerging adults reported alcohol use in the past year, with 13% reporting alcohol use disorder, 6.4% opioid misuse, 0.4% opioid use disorder—as well as 11.4% daily or near daily cannabis use in the past year, and 4% cannabis use disorder [23].

One of the reasons why emerging adults may use AOD is the influence that occurs in their social networks. Social networks are naturally occurring groups of people that can be characterized in terms of their composition (the quantity and types of network members) and structure (the relationships between network members) [24]. Social networks play an important role in the development of (and recovery from) substance use disorders. Network characteristics have been found to mediate change in the alcohol use of college students [25] and 12-step program participants [26–29]. Emerging adults may be particularly susceptible to influence on risk behavior from their social networks. Emerging adulthood is a period of social transition in which peer influences increase whereas family influences decrease [30]. Emerging adulthood is also marked by increases in risky behavior, including increased AOD use [19, 31]. Together, these processes create normative pressure towards increased risk taking among emerging adults. Urban AI/AN emerging adults are likely to have complicated network influences as they move between several different social worlds, including AI/AN peers and family in the urban areas where they live, non AI/AN urban network members, and AI/AN extended families living in rural, reservation areas [32].

To date, there are only a few studies that analyze the role of peer networks in AOD use among urban AI/AN adolescents or emerging adults. Earlier work indicates that urban AI/AN adolescents are often socially isolated within school networks or are tied to less cohesive school-based social groups, which can increase risk for AOD use [33, 34] To date, however, social network research on AI/AN youth AOD use is sparse, despite strong findings linking social networks and AOD use in other at-risk adolescent populations [33, 35]. There are no social network studies of AOD use among urban AI/AN emerging adults, and no intervention studies for urban AI/AN adolescents or emerging adults informed by social network analysis [36], despite the key role networks can play in triggering AOD use [37–41] and in discouraging AOD use and increasing resilience among urban AI/AN adolescents [35, 42].

Furthermore, there are few evidence-based AOD interventions for urban AI/AN people [15, 43], and none that address social networks explicitly. Studies with AI/AN adolescents, young adults, and adults have shown that one evidence-based treatment, Motivational Interviewing (MI), is viewed as closely mirroring AI/AN traditions, such as healing or talking circles [44, 45]. MI is also perceived to be culturally appropriate for AI/AN individuals because MI focuses on building resilience, creating positive change, and is nonjudgmental [46, 47]. Recent work has integrated the use of MI with social network feedback through personal network visualizations [48–51]. A randomized controlled trial demonstrated beneficial effects of this Motivational Network Interviewing (MNI) intervention on readiness to change, abstinence self-efficacy, and substance use [52], as well as beneficial changes to social networks [50], with an ethnically diverse and impoverished urban population of adults who reported substance use and have experienced homelessness and are transitioning into a housing program. The MNI approach was recently adapted for delivery to urban impoverished emerging adults experiencing homelessness transitioning into housing, [51] and this approach was found to be acceptable and feasible [53].

The current study presents results from an evaluation of an adaptation of the MNI for delivery to urban AI/AN emerging adults for inclusion in a substance use prevention intervention. The overall intervention, named “Traditions and Connections for Urban Native Americans” (TACUNA), is an adaptation of an existing culturally centered intervention for AOD use among urban AI/AN adolescents [43]. Details of the adaptation of TACUNA, including details of the process of incorporating community input to inform the adaptation, are available elsewhere [54, 55].

The adaptation and evaluation process of the MNI consisted of two phases. First, 13 two-hour in-person focus groups were held with AI/AN emerging adults ages 18–25, AI/AN parents, and providers of health services for AI/AN emerging adults. The focus groups were designed to elicit feedback on several components of the proposed TACUNA adaptation, including social network visualizations. Focus group transcripts were analyzed to inform workshop materials. Second, a pilot test of each of the three TACUNA workshops were held with AI/AN emerging adults ages 18–25.

The current paper presents a rapid analysis [56] of qualitative data generated to achieve two aims. The first aim describes reactions from focus groups to the core components of the MNI. The second aim describes reactions of urban AI/AN emerging adults to social network visualizations during a pilot test of workshop materials. Four research questions guided this analysis. First, would focus group participants find incorporation of social network visualizations into the culturally centered workshops acceptable, useful, and interesting? Second, what challenges to using this approach would they identify? Third, for urban AI/AN emerging adults seeing visualizations of their own social networks, what information would they notice about networks? And fourth, how would the participants react to the inclusion of social network visualizations into group MI sessions in the pilot test of the workshops?

This study describes the development of the first social network-based intervention to target AOD use among urban AI/AN emerging adults and the first adaptation of the MNI outside of the homelessness context. This is also the first attempt to integrate social network visualization feedback for delivery in a group MI format. Finally, the current study documents the first adaptation of MNI for delivery in a fully virtual group setting.

## Methods

### Sample and recruitment

Focus group recruitment occurred in three urban areas of California (North, Central, and South) with a purposive sample of urban AI/AN community members (parents, providers, and emerging adults). Eligibility for emerging adults and parents included self-identification as AI/AN and residence in an area outside of a reservation or tribal land. Focus groups were held between November 2019 and February 2020. Providers were eligible based on having experience treating AOD among AI/AN emerging adults; providers did not necessarily self-identify as AI/AN. The project team collaborated with a community organization, Sacred Path Indigenous Wellness Center (SPIWC), to recruit focus group participants through flyers at community events across California and word of mouth. Focus group participants were offered a $50 gift card for their two-hour time commitment. Similar recruitment procedures were used to recruit emerging adults for the 3-hour virtual workshops, which took place in July and August 2020. Pilot workshop participants received $100 gift cards. Focus groups were moderated by five different project team researchers–including 2 who are AI/AN (Inupiaq and Wahpeton Dakota)—representing a mixture of disciplines (cultural anthropology, clinical psychology, addiction psychiatry, and health policy and management). Prior to starting each group discussion, group moderators introduced themselves as members of the research team and informed participants of their rights as voluntary participants in a research study. All recruitment materials, data collection, informed consent, and analysis plans were approved by the lead author’s Institutional Review Board.

### Data collection

**Fig. 1:**
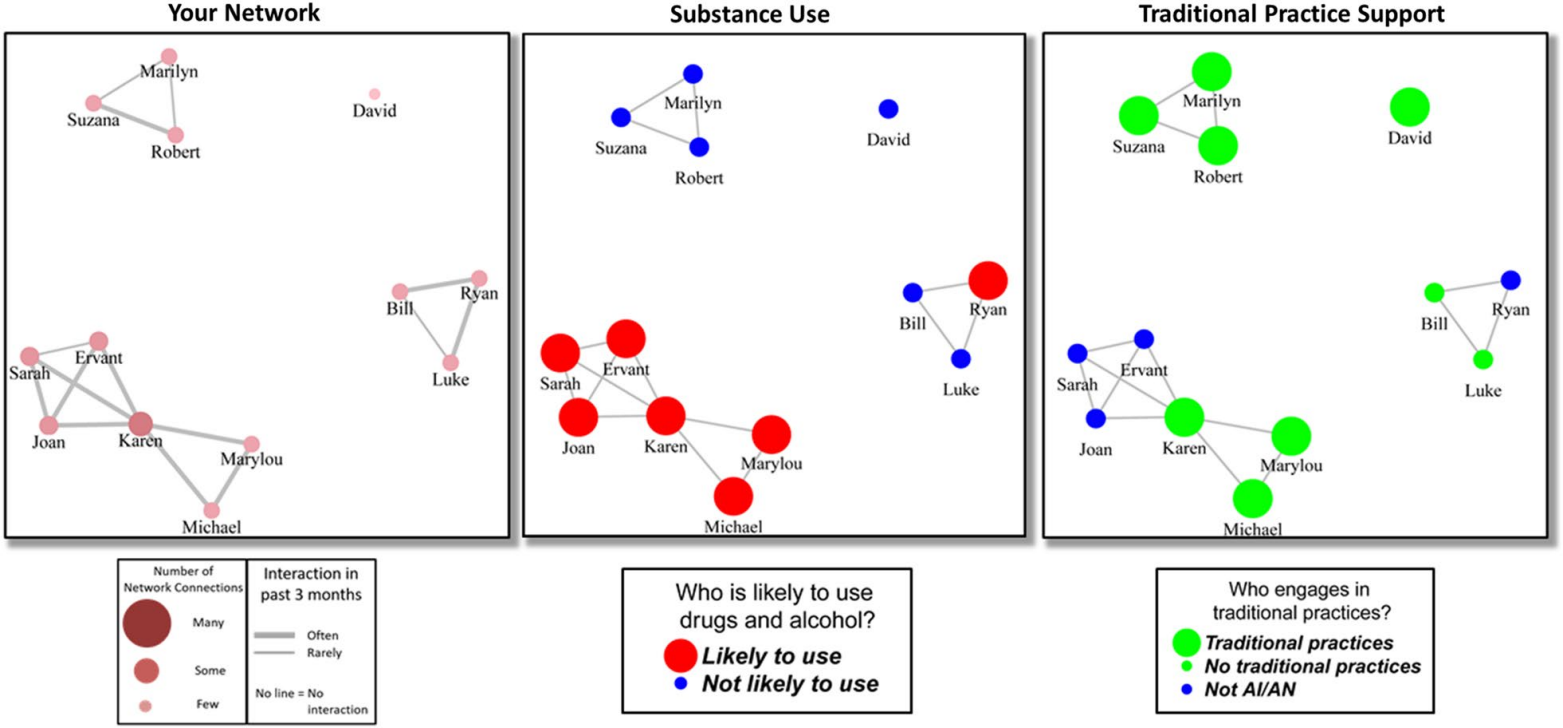
Hypothetical network visualizations provided to focus group participants. Network visualizations were generated with hypothetical data entered into EgoWeb 2.0. Example network members are represented by circles (nodes), labeled with example names, and lines between nodes represent members who interacted with each other in the past two weeks. Placement of nodes in two dimensions for each graph was generated using the “Fruchterman-Reingold” layout algorithm in the R package “igraph”. The “Your Network” graph on the left shows the names of people the participant reported interacting with in the past two weeks and highlights the centrality of nodes by calibrating node size and color with number of connections for a particular node (degree centrality), and line thickness with the participant’s rating of how frequently the two nodes interacted. The middle graph labeled “Substance use” shows larger red nodes for people who the respondent rates as likely to use AOD in the next two weeks and smaller blue nodes for those who are unlikely. The right-hand graph labeled “Traditional Practice Support” shows larger green nodes for people who engage in traditional practices, and smaller blue nodes for people who do not

Focus Group discussions were designed to present participants with proposed workshop content and prompt discussions about what content and materials participants liked, did not like, and what they thought should be included or excluded. Focus group data analyzed for the current study come from discussions of social relationships and reactions to example social network visualizations that the project proposed to include in intervention workshops. Appendix provides details of how focus group moderators introduced focus group discussions and specific prompts used to lead an initial discussion of social relationships (e.g., healthy relationships, the pathway between social relationships and opioid use) for each of the 13 groups. After this general discussion of social relationships, participants were then shown three example network visualizations from a hypothetical participant’s personal network data. Figure 1 depicts the network visualizations provided to focus group participants in a handout. The three diagrams highlight three aspects of one person’s personal network: (1) connections among network members, (2) AOD use by members of the social network, and (3) AI/AN identity and traditional/cultural practice participation by members of the social network. After providing the handout to the focus group attendees and briefly explaining the diagrams, group moderators encouraged discussion about how social networks influence both (a) opioid, alcohol and cannabis use and (b) traditional practice participation among urban AI/AN emerging adults. Participants were asked to contribute to the discussion in response to prompts about (1) how friends and others can influence healthy and unhealthy behaviors, (2) how peers can influence their friends in positive and negative ways, (3) how some people have many connections while others are socially isolated, (4) how AI/AN emerging adults are influenced to use opioids, alcohol or cannabis, and (5) how AI/AN emerging adults find support to participate in traditional practices, especially in an urban environment where they may be disconnected from others who share their cultural backgrounds. In addition, participants were asked to comment on their reaction to the use of the network diagrams in a proposed workshop.

A rapid analysis [56] of focus group transcripts informed development of workshop materials. For example, focus group feedback was utilized to create the facilitator protocol for viewing the social network diagrams for each group MI workshop. After a draft of workshop materials and protocol was completed, we held a pilot test of the three workshop sessions with a new group of urban AI/AN emerging adults to assess participant reactions to viewing and discussing personal network diagrams in a group format. Pilot sessions were held virtually due to the COVID-19 pandemic and social distancing orders, which emerged a few weeks after the final focus group. Therefore, pilot workshop sessions also provided a trial run of procedures for presenting and discussing personal network visualizations in a virtual setting.

Prior to the first scheduled workshop session, pilot test participants (N = 12) were provided with links to an online survey containing a series of structured survey questions. The survey was programmed using EgoWeb 2.0, which is open-source survey software customized for social network data collection and visualization and was customized for use in social network interventions. Structured questions included the primary components of a personal network data collection interview [57, 58]. First, participants were prompted with a “name generator”, to list names of network contacts with the following prompt:

*“First, think about the people you have talked with the most over the past three months, either in person or over the phone, or by texting, emailing...things like that. Please type names of 15 people who are at least 18 years old. You will be asked questions about each of these people in the following screens. Do not enter full names. You can use their first names, initials, nicknames, or some description that you will remember during this session and next time.”*

Next, participants were asked a series of “name interpreters” regarding the characteristics of each person. They were asked to (1) identify which of the people they named identified as AI/AN and participated in cultural or traditional practices and (2) identify who was likely to engage in heavy drinking, regular cannabis use, or taking other drugs such as opioids to get high. Participants were also asked “relationship interpreter” questions, where they evaluated each unique pair of network contacts and responded if the two people knew each other and if they interacted recently. Responses to name and relationship interpreter questions were the raw data used to generate network diagrams immediately after entering responses into software, EgoWeb 2.0. As participants viewed each diagram online, they were provided with a text box and asked to write reactions to what they saw. Prior to the pilot workshops, the diagrams were compiled into one PDF file for each participant. We then provided a link to each participant for their personal PDF file located in a secure file sharing site during workshop sessions. Participants were able to see their own network diagrams but not the diagrams of other participants. Moderators also did not view diagrams of any participant during the workshops, and participants were told not to discuss names of people in their networks but to talk more generally about people in their network.

Workshop moderators (2 members of the research team trained and experienced in clinical psychology, Motivational Interviewing, and addiction psychiatry) led a guided discussion of social relationships and how they can positively and negatively influence behaviors, during which workshop attendees were prompted to view their diagrams and discuss their own social networks (without mentioning any of the specific names they saw on their diagrams). Specific questions and probes included, (1) what they noticed about each of the graphs, (2) what types of people they could identify in their own networks, (3) who was missing from the network and might be someone to add in the future, and (4) how the network of interconnections influenced AOD use and engagement in traditional practices. Workshop moderators also prompted discussion of general network factors that contributed to healthy relationships among one’s families, friends, and participation in traditional practices. In one workshop, after a discussion of the social networks, participants were also asked to rate their willingness and confidence to make changes to their networks and to discuss why they chose their level of willingness and confidence to change.

Once the workshop sessions were completed, the moderators logged out of the virtual session and another member of the research team joined to lead a discussion of the workshop experience among the pilot test attendees. Discussion lasted approximately 45 min after each of the three workshops. Participants were asked to comment on what they thought of the social network diagrams and the discussion of social networks, and what suggestions they had for improvement.

### Analytic plan

The methodological approach to analyzing focus group and pilot session transcripts and notes was a rapid analysis [56], team-based [59], applied thematic analysis [60] similar to other intervention development studies informed by analysis of qualitative data [43, 49, 53, 61]. The approach relied on a combination of multiple data collection methods, triangulation during analysis, and iterative team discussion and revision of analysis results and conclusions. The research team selected this approach to efficiently guide the development and revisions of intervention materials informed by the themes related to social network diagrams that emerged from analysis of qualitative data.

We analyzed data from focus groups and pilot sessions by iterating between an inductive, grounded theory approach [62, 63] and a deductive, content analysis approach [64]. Focus group and pilot test sessions were audio recorded and transcripts were analyzed with open-coding [62] using qualitative analysis software to identify emergent themes participants discussed when viewing social network diagrams. Transcripts were de-identified and uploaded into Dedoose, a collaborative software platform used for qualitative data management and analysis. The analysis followed an iterative inductive and deductive analysis approach. Prior to open coding, the lead author defined codes to capture text segments related to the discussion of network visualizations based on the structure of the focus group and pilot interview guides. Next, a co-author applied these codes to the transcripts. Text segments from this initial coding were exported as.csv files and managed in Microsoft Excel. The lead author used an inductive technique for identifying themes in text [65] by sorting the text segments into distinct categories of comments about the visualizations and use of them in workshops.

The lead author also conducted a similar analysis of text entered by participants into open text boxes in the EgoWeb 2.0 system prior to the pilot workshops as they initially viewed their network diagrams. These text segments were exported from EgoWeb 2.0 into.csv files and opened with Microsoft Excel for inductive coding. The codes that emerged from this open coding process were then applied to all text segments using content analysis [66], which is a method of text analysis often employed to quantify qualitative data generated with responses to open ended survey questions [64]. We calculated code frequencies to assess prevalence of the themes across participants [67].

## Results: focus group discussions

Table 1 presents demographic information for focus group participants. Five main themes emerged from focus group discussions. Participants discussed (1) *feasibility* of the inclusion of social network visualizations into the workshops, (2) *acceptability* of this format for prompting discussions of social networks, (3) *relevance* of this approach to urban AI/AN emerging adults, (4) *usefulness*, and (5) *challenges* that may arise limiting the benefits of this approach. Table 2 provides exemplary quotes illustrating each of these themes.

**Table 1:** Focus group characteristics

Focus group demographics (N = 91)			
	Young adults (N = 32)	Parents (N = 25)	Providers (N = 33)
Age range (mean)	18–25 (21.5)	27–78 (46.5)	23–72 (48.9)
Sex N (%)			
Male	10 (31%)	4 (16%)	7 (21%)
Female	22 (69%)	21 (84%)	26 (79%)
Race N (%)			
American Indian/Alaska Native^a^	22 (69%)	19 (76%)	19 (58%)
Mixed heritage (AI/AN plus other)	10 (31%)	4 (16%)	6 (18%)
Other (White, Hispanic, Black)	0 (0%)	2 (8%)	7 (21%)

^a^Tribal affiliations are not specified to protect tribal confidentiality [68]

**Table 2:** Themes and illustrative quotes for focus group discussions

Theme	Type of participant and participant quotes
Feasibility	• [Emerging Adult:] “I feel like it’s a pretty easy system to understand, especially when it’s laid out side-by-side…I feel like it’s like really clear…And a good way of kind of thinking through those things” • [Parent:] “Yeah, I like it. I like it how they just give you the basics, the questions. They're answering our basic questions and then the program…separates their social network for them. And then it’s easy to follow” • [Emerging Adult:] “I feel like it’s still a little confusing, just a tad bit”
Acceptability	(Discussions of similar community led social network mapping exercises) •[Provider:] “We did an activity like this with some transitional-age (youth). And what we did was…talking circles…to really kind of identify their social network” • [Provider:] “Each person got a big poster paper…And what we were trying to do really was create a support system for who was there. So, our goal is…to understand we were there in a good way and trying to create positive connections” • [Emerging Adult:] “If it was a strong connection, there was like a solid line. And then if there was like, I know them, they're there all the time, but I don't really talk to them, it was like a dotted line and stuff like that…they each did their own. And then they discussed it at the end”
Relevance	(Discussion of interest in seeing network diagrams) • [Emerging Adult:] “I would be curious to see how many people…use [substances] in my circle” • [Emerging Adult:] “(It) would kind of make them think of…their life choices” (Discussion of relevance of the example network diagram) • [Emerging Adult] “Yeah, big time. I think especially having them right next to each other. It makes me think about, especially …with the whole “likely to use”, her being the center of it all, but then also engaging in traditional practices, just wanting to figure out more about her and why that is and how that works….I think it makes me want to know more about these people and their relationships and how those two things do interact with each other, because it’s interesting that you can go one of two ways almost in both of those, that there’s all these different splits that you can take and how knowing other people would affect that” • [Emerging Adult:] “I’m actually going to say this is actually pretty true for me, which is creepy. Because I have no network, basically…I really don’t like to interact with people…I stay sober…And I’m super into traditional practices and ceremonial ways and stuff” • [Emerging Adult:] “Well, I feel like I’m a “Karen” because I go out a lot. And I have like my group of friends that I go to music festivals with or raves • [Emerging Adult:] “Is it talking about in our substance use…the people that we’re likely to communicate with mostly likely are going to be using…on a regular basis? Because that’s totally true…I don’t really associate with anybody that’s sober…when I’m high, I really don’t. I mean, I don’t want to be around people who are sober. So, that kind of makes sense” • [Provider:] “So like for me, the center one with the big red circles, I kind of see that as like the home environment and the community. So, let’s just say you take one of those people out of that environment, right. They go to get help, substance abuse help. And the thing is that nothing is really changing there in the community in the home. And you got somebody over here getting well or you know trying to make changes. And for a young adult, most of them that I come across are not self-sufficient. So, they go back to the same place. And so you have a continuous cycle that’s happening"
Usefulness	(Q: Would the diagrams help participants think about their choices?) • [Parent:] “It’ll make them think…maybe Suzy and Johnny aren’t the best people to be with” • [Emerging Adult:] “(Yes) if it was drawn out for each individual person and they were serious about considering becoming sober or improving their life in any way, shape, or form…Like these are like the people around me (that) it’s really not in my best interest to be hanging around” • [Emerging Adult:] “The social networks was a really good idea, and the diagrams…helped visualize how your social network affects you. But I also think maybe we could talk about…parental figures or older cousins that you might see or other family members…someone who may be an alcoholic or someone else who may have a drug addiction. ‘Cause it’s not just like your peers, your friends that you’re hanging out with, it’s also like your family figures that are important in your life” • [Provider:] “Maybe I need to work on this relationship with this person because this person isn’t doing it…maybe I need to focus in on that and maybe connect with that person a little bit more • [Parent] “If the child…put down the people that are in their lives…then they could start highlighting, if they had a problem, who could they go to?” • [Provider:] “I just want to reiterate that seeing it on paper is actually a good idea… because for somebody like me, you know, I’m just trying to think, okay, who’s using, who’s not and trying to think of the whole network. Well, if you put it on paper you can see, okay, well, this is my network and for like guys in general, they're very visual” • [Parent:] “I think this would really help with young adults just because sometimes they're displaced out of their homes or they're growing into adults. And they're leaving their homes and their social networks are changing… you'll show them how they're moving and how they can in the future move” • [Provider:] “One of the conversations we had about staying clean is that more than likely you’ll lose a lot of those friends as you move forward in your sobriety. So, this really is a good way to put into perspective because it’s a visual” • [Parent:] “They could start eliminating and choosing a different, alternate, like getting a different result, if you're eliminating these negative people, your result's going to be a better chance that you're going to not repeat the same mistakes over” • [Provider:] “It could say what are some friends and relatives I shouldn’t be around and then do you have a choice not to be around them. I mean, if your mother is the one that’s using substances, you don’t really have a choice. And so, determine, like, where are my strengths, where do I have choice? Where can I exercise choice? You know, so they can get used to that habit. I have a choice to select people sometimes, sometimes I don’t” • [Provider:] “I like it because I don’t think we teach our youth refusal skills enough or critical thinking skills enough… because we’re so community-minded, Native people, sometimes we don’t rate our relatives or rate our friends and in this world we do need to say, “Hey, we love Uncle So-and-So, but we don’t go hang out at their house because they always have alcohol.” And to get our youth to start thinking that way…our traditional values are that we love everybody, and we value everybody, and we lift everybody up, but there are some people you don’t want to hang out with if you’re a young person and they’re going to be exposing you to alcohol or other unhealthy behaviors. So, I’d like it because it sort of gets into refusal skills and critical thinking skills” (Discussion of using diagrams to track changes over time) • [Parent:] “It changes over time like a progress chart…It can be good because it can show…how you've shifted and who you interact with” • [Provider:] “It would be cool for them to see how it changes, because they'll be able to look at it over time and that can be really powerful…They'll see, oh look, I made these changes in my network and because of that, I'm not using as much”
Challenges	• [Provider:] “How do we do that in a way where it’s like that diagram isn’t going to immediately shut people down…I was looking at that and I’m like, who’s actually not using substances? And a lot of my dots would be red, you know what I mean? So, I’m like thinking to myself, as a young person, I would feel like, dang, I don’t even have anybody in my network—I would isolate myself from my whole family if I was to…try to transition” • [Provider:] “So when you say, ‘Hey, bring your healthy network… ‘Well, yeah, I don’t have anyone, so I'm just not going to come at all.’…Because obviously if they’re at-risk and within this group, they’re not going to have those healthy connections” • [Emerging Adult:] “I think I've witnessed… some people…stick with that group because that's like their only friends…try to avoid those people, but it can be difficult” • [Emerging Adult:] “I think a barrier, too…There’s elders that are also in their addiction…So, it’s hard to have both…others within the family could make you feel like that there’s almost like a betrayal. Now, you’re leaving us. You’re better than us”

*Feasibility.* Table 2, row 1, provides quotes illustrating the “Feasibility” theme, which provides comments endorsing the use of social network visualizations in the proposed workshop intervention sessions. Participants across each type of focus group (providers, parents, and emerging adults) commented on the feasibility and acceptability of using network diagrams in a workshop directed at preventing opioid, alcohol, and cannabis use among urban AN/AI emerging adults living in urban areas. Upon viewing the diagrams with their associated descriptions, many provided simple confirmations when asked if the visuals made sense to them (e.g., “Yeah”). Some comments further explained that the diagrams intuitively made sense and were easy to follow and understand. Only a few comments indicated some confusion when viewing the diagrams.

*Acceptability.* Row 2 of Table 2 provides quotes illustrating the “Acceptability” theme. Across each group, participants indicated that the proposed use of social network visualizations as part of an intervention with urban AI/AN emerging adults would be acceptable in their communities. Several discussions in provider focus groups emphasized this point by recounting that they already did similar exercises. In one group, a provider described using a similar technique to engage with young adults about their social networks by having them draw these networks first to facilitate discussion that would lead to social network changes. In a different group, an emerging adult discussed participating in a similar exercise and described how it involved learning about social ties by drawing different types of lines between people to represent strength of relationships.

*Relevance.* Row 3 of Table 2 presents quotations illustrating the theme of “Relevance”, which summarizes discussions about how viewing diagrams would be interesting for urban AI/AN emerging adults to view and talk about in the workshop. Many comments from emerging adults in the focus groups suggested that they would find it interesting to view their own social networks if they were part of a workshop. Some explained that seeing network diagrams would be an enjoyable part of the workshops because they would be able to understand how their networks functioned, and that would help them better understand the role of AOD use and traditional practices in their lives. Several participants commented how well the example diagram (Fig. 1) depicted social situations that were familiar to them. One affirmed that viewing the three diagrams together, especially following a particular individual (such as the node labeled “Karen” in Fig. 1) across the three diagrams, would increase the curiosity of workshop attendees. Although the focus group diagrams depicted a fictional network, several comments suggested that viewing the diagrams generated thoughts of specific network interactions participants had experienced or had observed in their own networks. Several emerging adults pointed out which labeled node on the diagrams best fit their own social situation. For example, one emerging adult identified with a network member (labeled “David”) who did not engage in AOD use, engaged in traditional practices, and had no connections to the rest of the group. On the other hand, another emerging adult personally identified with the most connected member of the network because she liked to socialize. Another emerging adult noted that from personal experience, the clustering together of substance using nodes in the AOD diagram made sense because she avoids sober people when she is using substances. A participant in the provider group also noted that the demonstration diagrams represented real network dynamics for AI/AN emerging adults who reduce AOD use by removing themselves from dense networks of use in their home communities but are never fully separated from this network influence.

*Usefulness.* Row 4 of Table 2 presents quotations illustrating the theme of “Usefulness”, which captures the discussion related to how incorporating social network visualizations into a behavior change intervention help people positively change their behavior. Discussions across each type of group confirmed the usefulness of visualizing social networks in addressing AOD use and engaging in traditional practices. Participants discussed ways that viewing the diagrams could help identify network members who should be avoided, especially if they wanted to make healthy choices, including avoiding family members who engage in AOD use. Participants also commented that viewing diagrams could help identify people they should connect with and spend more time with because they could provide support.

Several participants emphasized that depicting networks visually would help people think through decisions about interacting with people in the network who are engaging in AOD use or not and this would especially help those who are visual learners. Providers emphasized that this was especially important for those who transition from use to sobriety because this transition often results in losing friends. Some comments from parents emphasized how viewing the diagrams could help AI/AN emerging adults cope with social network transitions that occur naturally, such as transitioning to living on their own and the effects this has on their social networks. In addition, parents stressed the value of viewing the diagrams for making active changes to the network during this period of social transition. A member of the provider group suggested that the network visualizations could help emerging adults sort out who in their social networks they did or did not have a choice to avoid if they are trying to reduce substance and how the influence of other people may affect their behavior. Another comment from the provider group suggested that viewing network visualizations could help build skills for avoiding negative influences from social network members even if they are family members. Finally, several parents and providers suggested that the visualizations would also help document how successful emerging adults are in changing their networks over time if they were making changes and wanted to see evidence of the progress they were making.

*Challenges.* Row 5 of Table 2 presents quotations illustrating the theme of “Challenges”, which summarizes discussion about difficulties that may arise when attempting to change social networks. Although much of the discussion of the social network visualization tool focused on acceptability and usefulness, discussion also touched on challenges in addressing problems embedded in social networks. Emphasizing that AOD use may often be common in families, on reservations, and among peer groups, some participants cautioned that attempting to disconnect from those who use substances could lead someone to disconnect from their social environment completely, or that the fear of being isolated might prevent someone from being able to avoid AOD use in the network. Providers discussed the challenge of giving social feedback about the use of substances in a person’s network because it can be difficult to disconnect from people who use substances and still maintain supportive social connections. Providers also commented on the difficulty of replacing unhealthy network members with healthy ones because some networks may be saturated with unhealthy influences for those who are “at risk”, and this may be discouraging. An emerging adult similarly commented that it would be challenging to change a social network that is full of people who use AOD, making it rare that someone detaches from the influence of a friendship group that is dominated by use. Another emerging adult also emphasized the challenges of transforming friendship networks from healthy to unhealthy, and added that this was challenging because respected members of community were sometimes using AOD, making it difficult to disengage from them without sending a message of disrespect.

## Results: reactions to personal network diagrams online

**Fig. 2:**
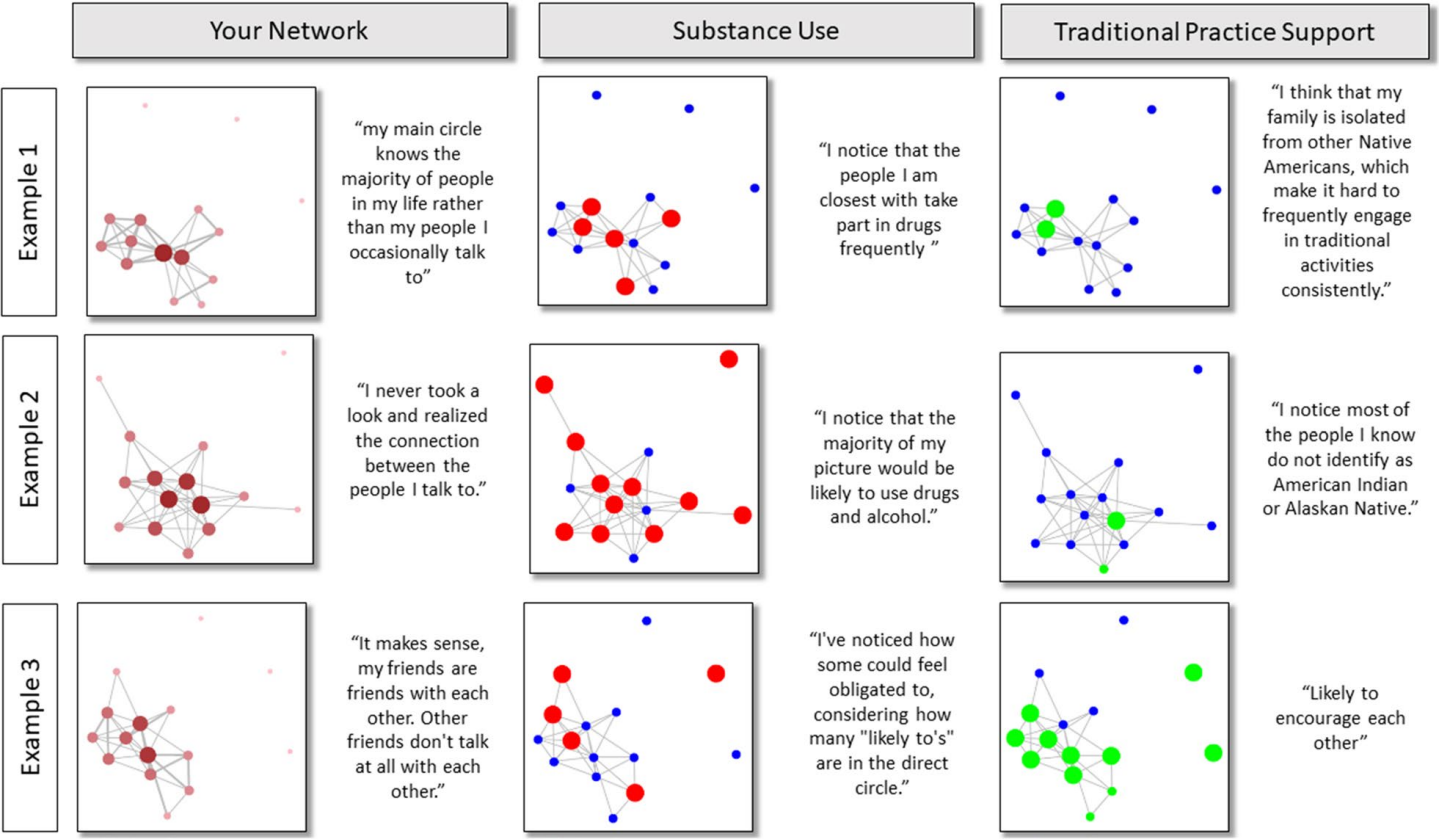
Network visualizations of 3 pilot workshop participants and text comments entered by participants into EgoWeb 2.0. Network visualizations were generated from pilot participant network data using the same layout, node size, node color visualization processing steps as Fig. 1. Participants viewed these visualizations directly in EgoWeb 2.0 after entering responses to questions about their networks. Node labels displayed in the EgoWeb 2.0 diagrams viewed by the pilot test visualizations have been removed. Text associated with each visualization is a verbatim response entered into a text box by each participant when viewing the diagram. Participants were prompted to enter text with the following text: (Your Network) “Take a look at the picture and think about what makes sense to you about the picture. Also, what is something you did not realize about your social network before looking at the picture?”; (Substance Use) “What do you notice about your picture?”; (Traditional Practice Support): “What do you think about how these different types of people are connected with each other?”

Twelve emerging adult participants who had not participated in the focus groups attended three pilot workshops. Prior to the workshops, participants completed on-line personal network interviews whereby they responded to structured questions about their networks and were shown personal network visualizations. Each of these participants responded to an open-ended question on each screen with each of the three figures (e.g. “What do you notice about your picture?”). Figure 2 provides examples of the set of 3 visualizations and comments provided. Open coding analysis of the pattern of responses for all 12 pilot workshop attendees identified three primary themes for discussions that occurred during the workshops. Table 3 provides detailed summaries of these themes, a count of the number of participants who made a comment that fit the thematic pattern, and exemplary quotes that illustrate each theme. First, most participants (8 of 12) wrote comments about how they gained (1) *new insight* about their network, including a new understanding of how network members were connected to each other, their use of AOD, or their engagement in traditional practices. A third of participants commented that the visualizations (2) *made sense*, confirming what they already knew about their networks. Every participant wrote at least one response indicating an intuitive understanding of (3) *network concepts* commonly addressed in social network analysis. Over two-thirds (10 of 12) of participants wrote comments about their *network composition* (the types of people in the network), two-thirds (9) wrote comments about *network structure* (the interconnections among network members), and half (6) wrote responses that were about the *characteristics of relationships* among network members or between the participant and their network contacts.

**Table 3:** Description of themes identified in the comments entered by pilot test participants when viewing their personal network visualizations

Theme	Description	Example quotes
*New insight* (8/12)	This theme includes comments about the participant gaining a new understanding of how network members were connected to each other after viewing their graphs. Comments described insights related to (1) interconnections among network members, (2) insights related to what they noticed about AOD use among members of their network, and (3) insights related to engagement in traditional cultural practices	•“Something I did not realize was how some members of my social network hardly know others or do not really know each other that well. Or how close some are compared to others, now that I see it laid out in front of me now.” •“What I didn’t realize is how connected everyone is even though they don’t connect all the time. It makes me realize how I might have two lives or separate the people who are in my life.” •“They are all connected. I did not realize we're all interconnected” •“I notice that the people that are closest to me are the ones who use drugs.” •“I think it is interesting to know who does not share or shares my same cultural traditions.”
*Made sense* (4/12)	The theme represents a pattern of responses from participants about how the visualizations made sense to them because the visual features were consistent with what they already thought about their network	•“It makes sense that majority of the individuals have connections in some way since I communicate with some individuals which can lead to them sharing with the others and then the others reconnecting with me.” •“It makes sense, my friends are friends with each other. Other friends don't talk at all with each other.”
Network concepts: network structure (9/12)	The theme includes a pattern of descriptions of networks that were consistent with concepts used in social network analysis to characterize interconnections among network members	•“Lots of people I know, know others” (density) •“They are all connected” (density) •“That was the only reason most of these people had come in contact with another, because of me. Other than that, the people listed that are related had their own contact and connections.” (centrality) •“My main circle knows the majority of people in my life rather than my people I occasionally talk to” (centrality) •“Those who I think may use them had a lot more connections to everyone else compared to those who I do not think would use.” (subgroups) •“I think that my family is isolated from other Native Americans, which make it hard to frequently engage in traditional activities consistently.” (components) •“The AI/AN people I know are self-contained from the rest of my network.” (components) •“It makes me realize how I might have two lives or separate the people who are in my life.” (bridging)
Network concepts: network composition (10/12)	The theme includes a pattern of descriptions of networks that were consistent with concepts used in social network analysis to characterize the types of members of a network. Comments in this theme included mentions of the quantity of people who used or did not use AOD in the network. Also included were respondent comments about high or low proportion of network members who engaged in traditional practices or shared a cultural identity with the participant	•“I notice that the majority of my picture would be likely to use drugs and alcohol” •“I notice that I have more members of my social network that I do not think will use drugs or alcohol than I do for who I think may use them.” •“I've noticed how some could feel obligated to, considering how many “likely to’s” are in the direct circle.” •“I think it’s interesting that each one participates in traditional events in different ways” •“I notice most of the people I know do not identify as American Indian or Alaskan Native.” •“The people that are the red big circles are the same ones I'm in constant contact with that would make sense of being able to have an influence or impression on me.”
Network concepts: characteristics of relationships (6/12)	This theme included comments about the quality of relationships participants had with network members and/or network members had with each other. Although not explicitly visualized, respondents identified patterns of relationship characteristics in the diagrams and noted the factors that tied certain individuals together more strongly than others. Relationship characteristics noted included frequency and recency of contact as well as feelings of closeness	•“The bigger red dots are the people I am in constant contact with.” •“There are some friends of mine that haven’t talked with one another in some time.” •“I feel like I have a stronger bond and understanding with my family that holds traditional values.” •“I notice that most of the individuals likely to use are young adults.” •“The blue circles are my older family members…that do not drink or party they are all over 35 + and are very involved in ceremony ways.” •“The others…are Indigenous young people under 25 who are all involved in cultural activities, but I think struggle with sobriety, depression, trauma etc.” •“Likely to encourage each other.”

## Results: pilot workshop discussions

Similar to focus groups, feedback discussions after the pilot workshops highlighted the *feasibility* and *acceptability* of using social network diagrams in a group setting to talk about relationships. Participants also discussed how seeing their network provided *new and important insights* into the relationships they have with people, and how these people may influence the choices they make. Table 4 provides exemplary quotes illustrating each theme that emerged from open coding of pilot workshop session discussions.

**Table 4:** Themes and illustrative quotes from pilot workshop discussions

Theme	Participant quotes
Feasibility	(Discussions of comfort with group discussions of social network graphs) •“Yeah, I was comfortable with sharing in that way.” •“We were just able to talk about it without having to say names, so I thought it was really good.” •“I like the level of anonymity that there was where the people leading the discussion were just kind of like, ‘Oh, who is assigned to which variable? Which groups are assigned to it?’ No names asked. The anonymity is still there, so I liked that. And there weren’t really any assumptions made by the people leading the discussion as far as I could tell.” •“The Native community is small. But…I’m actually surprised I don’t know anyone in here personally or see them around at events. So I think maybe possibly that’s why the conversation was a lot smoother.”
Acceptability	•“I personally liked it because we are who we surround ourselves with.” •“We know who we talk to but to actually see it that way was really cool.” •“Super interesting, new perspective of our social networks.” •“Well, it’s kind of a fun thing, ‘cause we don’t normally think about it, so it’s kind of fun, I think, to look at and see, ‘Oh, this is who I’m hanging out with and this is how they all go together.’” •“I was actually pretty surprised, because it got the exact people who actually use drugs, and I know they do…It was very accurate.” •“I kind of always knew but…it was never really in front of me, where it's like a proven fact…more clarity is what I gained from the map, which is really crazy because I never thought that it would be right in front of me, like physically… It’s not just in my head” •“Just lightbulb in my head, just filling out the survey… seeing wow, I do know people that harbor that duality of being in the traditional spiritual practices and being a user at the same time. And it's just that pull is constant, as well.” • “I think it was good, but there were some variables that I think were brought to light afterwards, after creating the social network…there’s a difference between people who moderately use substances and those who struggle with them…if the evaluation is simply amended to assign somebody either to moderate use or…problematic use or heavy use… that definitely would have helped me conceptualize and visualize how moderate and heavy substance use connects together.”
New and important insights	•“What I noticed with mine was that a lot of the people that I know only know each other or are connected because of me.” •“I noticed that everybody knew each other like through me or through…activities that we do, like pow-wows or ceremonies and stuff…except for two that were not Native. They didn’t connect with anybody. Everyone else was connected.” •“I noticed that I don't have…many friends that are outside of…ceremony or powwows…I was like, oh, dang, I need more friends of…different ethnicities.” •“I think mine was interesting to see because everybody in my network kind of had an interaction. And I think that's maybe just because of me being able to talk to them, having them talk to me and—kind of like a telephone effect. So that was interesting to see everybody was connected in some way…I didn’t really see—with the example that you showed, I didn’t really see any separate outside. So yeah, that was interesting.” •“A lot of people I know connect through sports or tradition, ‘cause that’s how it had been, well, for the past four years. That’s how I’ve been breaking up my, I guess you could say my friend group, ‘cause there’s sports, tradition, and school. So there’s a point where the substance abuse stops once you start getting into the traditional mix and then also where it begins and it's very…complex” •“Something that was pretty good is that…there was way less people likely to use and abuse drugs than people who are sober. I only had a few people in my network who were kind of in that area.” •“I liked it, because we could see different patterns and trends in our social networks and talk about them” •“I think the diagrams…and then while they were talking about it while it was right in front of us were the most helpful for me” •“I wouldn’t say I learned much new from it. I kind of already understood who used or didn’t use certain substances. But it was definitely grounds or a foundation for further discussion. Having it there was a very necessary tool to talking about what we did then. I would say it was pretty basic stuff when it was laid out before me. It was a nice visualization, but it was mostly tools for discussion thereafter. So I liked that.” (Discussing diagrams visualizing traditional practices and AOD use) •“It kind of like assumes that people that do traditional things don’t ever struggle or slip up with their sobriety.” •“Some people in my network…they participate in traditional practices in their culture, but they also have a habit of partying and being on the scene…So that was a huge eye-opener for me.”

*Feasibility*. Table 4, row 1, provides quotes illustrating the “Feasibility” theme, which describes comments from participants about their experience discussing their social network visualizations in a group and their comfort level during this discussion. Participants commented that including social network visualizations and discussion of social networks in the group workshops was highly feasible. They emphasized how the format provided sufficient comfort to discuss their networks in a group format. Importantly, participants mentioned that the emphasis on not mentioning specific names helped reduce concerns about this type of discussion. Participants mentioned that the virtual format of the workshop enabled mixing together participants who did not know each other before the session and this anonymity enabled conversation. Participants also noted the value of anonymity for encouraging discussion about social networks among members of the Native community, who may often know each other’s social networks and might be reluctant to discuss people known to other participants.

*Acceptability.* Table 4, row 2, provides quotes illustrating the “Acceptability” theme, which represents the pattern of comments from participants about their reactions to viewing their own social network diagrams. Most participants commented that viewing their own network diagrams during the workshop was a positive experience and helped them understand their relationships (e.g. “I liked it.”). Participants also emphasized that they enjoyed seeing how accurately the diagrams represented their social networks. Participants commented on how much they enjoyed viewing their own personal networks as it helped them see the potential overlap between substance use and traditional practices. Although pilot test participants made positive comments about viewing the diagrams as part of the workshops, they also provided important feedback about how to improve the presentation of network information, such as changing the wording used to differentiate AOD use among network members (“moderate” use contrasted with “heavy” use).

*New and important insights.* Table 4, row 3, provides quotes illustrating the “New and important insights” theme, which summarizes responses about things that participants did not realize about their social networks before viewing them for the first time. During pilot workshop discussions, participants elaborated on the comments they made when viewing their networks online. Insights included the apparent connectivity in the network and evidence of networks that bridged different worlds, such as those who did or did not engage in traditional practices. Other comments described insight into the ethnic composition of their networks, including a visible lack of ethnic diversity that their traditional practices diagram made clear. Participants also commented on how their networks were interconnected and contrasted their own network structure with the structure illustrated in the workshop’s example network. Participants discussed noticing different groups of individuals in the diagrams and how these groups were often related because of AOD use or Native identity. One participant drew attention to how the diagrams that separated AOD use from traditional practice engagement into two individual diagrams could give a misleading impression by implying that there is no overlap in these activities. However, other participant comments about noticing overlap in AOD use and engagement in traditional practices in their own networks suggested that this was self-evident when viewing the 2 diagrams side by side. Some comments discussed feeling encouraged that their networks included more people who were sober relative to those who used substances. Pilot participants also emphasized that viewing their networks enhanced the workshop discussions of how networks could affect both AOD use and engagement in traditional practices. Participants often identified viewing their social networks as their favorite part of the workshops, reporting that the conversations about the diagrams enhanced the workshop experience and gave them important insight into their relationships.

## Discussion

The current study presents empirical insights from participant feedback required to adapt an innovative behavior change intervention approach that combines MI with personalized social network visualizations for urban AI/AN emerging adults. This work is an important first step in developing interventions that directly engage urban AI/AN emerging adults about their social networks in order to encourage discussions of how these relationships may increase both risk and resilience and how to take steps to make changes in their lives if they were ready to do so. The MNI approach has been found to be acceptable to emerging adults experiencing homelessness, and has been successful in influencing positive substance use [52] and social network changes [50]. Prior to this study, the MNI approach has not been adapted outside of the homelessness and housing context. Thus, we conducted focus groups with urban AI/AN community members to inform the adaptation of the MNI social network intervention approach for urban AI/AN emerging adults. Focus group participants made many comments indicating that that social network diagrams were easy to understand, acceptable, highly relevant, and interesting to view and discuss. Some discussion also focused on challenges associated with changing networks, and how talking about their social networks could be potentially helpful in prompting and tracking positive network changes.

After developing workshop materials based on focus group feedback, we further assessed the intervention approach in a pilot test of the TACUNA workshops, which included discussion of participants’ social network visualizations. Pilot test participants described these discussions as acceptable, reported experiencing new insights about the role of social networks within their lives, and found that the visualizations made sense to them and that the inclusion of the social network diagrams in the workshops encouraged interesting and useful discussion.

Overall, findings suggest that incorporating discussions of social networks using visual aids may be a promising way to help urban AI/AN emerging adults identify how their health and overall well-being is influenced by their social networks. Key findings highlight the acceptability and feasibility of social network visualizations with this group. They also demonstrate the positive effects of these visualizations on AI/AN emerging adults’ understanding of how their relationships may influence the choices they make surrounding risk behaviors, such as AOD use, and protective behaviors, such as participation in traditional practices. Social network visualizations are typically used by social network researchers when analyzing data from research projects. However, comments across the focus groups, pilot test workshops, and write in comments from the network survey demonstrated a clear understanding of the information conveyed in these diagrams and repeatedly emphasized their potential usefulness in a prevention intervention.

Participants consistently voiced supportive comments about the meaning and utility of the diagrams for conveying information and encouraging behavior change. A range of different types of participants (parents, providers, emerging adults) expressed these views in three distinct settings with network diagrams presented in three different formats: in focus groups discussing hypothetical network visualizations, in self-administered online surveys displaying personal networks, and in pilot test workshops in a virtual group setting comparing insights that participants gained from viewing their networks. The pattern of comments across these formats indicated that the diagrams told intuitive, relevant, and important stories. In each case, individuals emphasized that discussions about the diagrams would increase emerging adults’ awareness of their social environment and help them evaluate how their networks affected their choices around AOD use and participation in traditional practices. Furthermore, participants indicated that the visualization could allow them to think through whether they wanted to make changes in their behavior, and help them determine the best way to do that if they were ready to do so.

There are few developmentally and culturally appropriate interventions addressing AOD use for urban AI/AN emerging adults that also incorporate evidence based treatment [15]; thus, this study addresses several critical gaps in the field. Prior to this study, the intervention approach of integrating MI with social network diagrams had never been used with AI/AN individuals. The focus group discussions established acceptability of this approach, and also indicated familiarity with using visualizations of social relationships as a technique for engaging with AI/AN emerging adults. Similar to other MI social network intervention research [48–52], pilot test results emphasized the usefulness of viewing and understanding social networks in relation to both risk and resilience, and showed that it is possible to do this in a virtual group format. Participants were primed to think about their networks based on the survey they completed, and it is notable that they felt comfortable reviewing their personalized network and discussing the relationships generally with the group without having to name individuals. This suggests that the group format was non-threatening and conducive to open discussion of social experiences, which is essential for conducting MI successfully in this format [69, 70]. Another important finding was discussion of the complex relationships that occurred as people in their networks may increase risk by using AOD, but these same people may also be protective by engaging in traditional practices. Discussion in the pilot workshops focused on specific actionable ways that AI/AN emerging adults could change their behaviors given this duality. This was a key point brought up numerous times and is something that providers must be prepared to address, particularly in urban settings where it may be difficult for AI/AN individuals to access traditional resources [71, 72].

As with many projects, the pandemic affected the original study design. However, we were able to successfully pivot from an in-person group format to a virtual group format, which was not only acceptable to urban AI/AN emerging adults, but was also considered a benefit as it enhanced anonymity, thereby increasing sharing and discussion. Furthermore, the virtual format enabled interactions with AI/AN emerging adults in other geographic areas, which also enhanced comfort in discussing social networks.

To our knowledge, no behavioral evidence-based interventions exist that incorporate the role of social network visualization for urban AI/AN emerging adults. Further research is needed to understand the effects of incorporating a motivational network intervention into behavioral interventions for AI/AN people. The insights gained from this study informed the final curriculum, MI facilitator protocol, and workshop materials for a randomized controlled trial [54], which is ongoing. If successful, results from this trial may help guide providers as personal network interviews and visualizations could provide a tool to discuss relevant social context, which can inform the development of behavioral health treatment plans to modify social connections to address challenges or enhance protective factors in their networks. In addition to demonstrating the potential for interventions with urban AI/AN emerging adults, findings suggest that the MNI may be a flexible approach that can be adapted and applied to other populations and outcomes. Positive reactions to the MNI and the discussions of the acceptability and feasibility of including the MNI in a substance use prevention intervention are similar to reactions of ethnically diverse participants in previous studies that addressed substance use in the context of homelessness [49, 53]. The use of visual aids by clinicians to help clients address social support has a long history in the field of social work, [73] and findings from this study coupled with previous evaluations of the MNI suggest that using personal network visualizations in clinical settings is a feasible, acceptable, and interesting way for clinicians to engage with clients about relevant social factors. The current study provides an empirical foundation for future work to further explore how the MNI may be adapted to other behavior and social change contexts.

*Limitations:* Although results provide support for acceptability, feasibility, relevance, usefulness, and understandability of this intervention approach, there are some limitations. First, participants in focus groups were a purposive sample of emerging adults, parents, and providers from Northern, Central, and Southern California and their opinions may not generalize to other populations. Another limitation to focus group discussions is that participants volunteered for the sessions after learning about the project through recruitment advertisements disseminated by community partners that serve the AI/AN community. Participants may have been more interested in the intervention than a similar group of people who did not volunteer. Further, participants were aware that facilitators of focus groups and pilot test sessions were members of the research team and may have felt uncomfortable offering criticisms of the intervention descriptions and content. Of note, focus group facilitators introduced the group discussion as an opportunity to develop and improve the intervention based on their feedback and frequently probed for discussions of content that should be changed or improved. Further, a different member of the research team conducted the feedback session for the pilot sessions, and the workshop facilitators were not part of this feedback session.

A limitation to the group format of the data collection is that it does not allow disaggregating individual responses. However, our findings were not limited to focus group responses only. Pilot test participants also provided individual comments online when viewing their social networks for the first time prior to the pilot workshops, and many of these comments re-iterated themes that emerged from the focus group and pilot test workshop data, demonstrating that participants independently recognized the key aspects of their networks and related them to their own lives. This “triangulation” of different data collection methods converging on a similar set of themes is a technique for enhancing validity in qualitative research [60]. Another limitation is that we identified themes by analyzing the full set of focus group and pilot test data; that is, we did not conduct tests to determine if we reached theoretical saturation. Therefore, we do not know if additional themes would have been generated with additional focus groups. However, the number of groups we conducted (13) was larger than the number of focus groups recommended to reach saturation (three to six) based on empirical studies [74].

## Conclusions

Despite limitations, findings provide empirical evidence that using personal network visualizations as part of a culturally-tailored, group MI, AOD prevention intervention for urban AI/AN emerging adults is considered feasible and acceptable to emerging adults and other AI/AN community members. While recognizing the challenges associated with making changes to social networks, focus group members agreed that the approach is relevant for urban AI/AN emerging adults and expressed support for the potential usefulness of engaging them in discussions of their social network through personal network visualizations. The reactions of pilot test participants to viewing and discussing their own personal networks reinforced focus group discussions. Participants described novel insights they gained into their social networks from viewing them online and enjoyed discussing what they noticed in a virtual, group MI format. Findings support continued development of behavior change interventions that address the social context of behavior through personal network visualizations.

Emerging adulthood is a time of increased AOD use risk due to social influence as well as increased independence from the family structure. Urban AI/AN emerging adults seem to experience particularly challenging social worlds and are often positioned between a diverse set of social influences that may affect their AOD use. They may also experience disconnection from cultural resources given the urban environment. Social network visualizations provide an important tool for navigating these complex social challenges and increasing protective factors for this population. The successful adaptation of the MNI for urban AI/AN emerging adults also suggests that the combination of MI and personal network visualizations may be a useful element of behavior change interventions for other populations and outcomes.

## Data Availability

Once collected, deidentified data from this study will be available from the corresponding author on reasonable request one year after all aims of the project are completed. Requestors of data will be asked to complete a data-sharing agreement that provides for (1) a commitment to using the data only for research purposes and not to identify any individual participant; (2) a commitment to securing the data using appropriate computer technology; and (3) a commitment to destroying or returning the data after analyses are completed.

## Appendix

In the next two hours or so, we will be talking with you about what you think about social relationships, Native American identity, and challenges you [your son/daughter/clients] may face in your community. We will also present information from a program that focuses on social networks, opioid misuse, and alcohol and other drug use. We will ask for your opinions about things you like or don’t like about the program and how we could make it better. You are the experts here. We would like your honest opinions, so please do not be afraid to speak up or to be critical.

Our past research has suggested that social relationships can be both helpful and harmful among your peers and in your community [for youth]. For example, some peers may be supportive while others may harass you, including making discriminatory comments about your AI/AN background. Also, some friends might help draw you towards healthy activities while others might draw you towards drug use or other harmful behaviors. Some relationships can be positive in many ways, such as relationships with family members, but sometimes they be a negative influence on harmful behaviors, such as drug use.

We also know that how your peers interact with each other is important. Peers who have a lot of friends can influence a lot of other people in positive and negative ways. Some peers might be positive influences, but they might hang out with a group of peers who engage in harmful behaviors or are negative towards people with AI/AN backgrounds.

We are trying to understand how social relationships among AI/AN people might affect opioid misuse, for example, and also how social relationships might affect participation in traditional activities, especially in an urban environment.

Finally, a big problem for some is that they feel isolated from peers and do not have many people they can turn to for support and do not have a group of peers they feel part of. This isolation sometimes can lead to opioid use or alcohol use.

How does this sound to you? Is it accurate or does it miss some important things? What else should we know about social relationships and how they affect your life, health, stress level, and happiness?

[Probe on issues mentioned above. How might they affect the choices people make?].

Also, it is not uncommon to feel lonely or isolated, even in the big city environment. How can feeling isolated affect your ability to have healthy relationships? How might substance use be involved with isolation for instance? Also, some Native people may feel less connected with other Native people in the urban environment. What is your perspective on feeling isolated or disconnected with others in the urban setting?

## Abbreviations

AI/AN: American Indian/Alaska Native
MI: Motivational interviewing
MNI: Motivational network interviewing
RCT: Randomized control trial
TACUNA: Traditions and connections for Urban Native Americans
AOD: Alcohol and/or other drugs
HEAL: The helping end addiction long term initiative
